# Quantitative proteomic analysis of aberrant expressed lysine acetylation in gastrointestinal stromal tumors

**DOI:** 10.1186/s12014-021-09322-0

**Published:** 2021-05-22

**Authors:** Bo Wang, Long Zhao, Zhidong Gao, Jianyuan Luo, Haoran Zhang, Lin Gan, Kewei Jiang, Shan Wang, Yingjiang Ye, Zhanlong Shen

**Affiliations:** 1 Department of Gastroenterological Surgery, Peking University People’s Hospital, Beijing, People’s Republic of China; 2 Laboratory of Surgical Oncology, Peking University People’s Hospital, Beijing, People’s Republic of China; 3 Beijing Key Laboratory of Colorectal Cancer Diagnosis and Treatment Research, Beijing, People’s Republic of China; 4 Department of Medical Genetics, Peking University Health Science Center, Beijing, People’s Republic of China

**Keywords:** Gastrointestinal stromal tumor, Acetylation, Proteomics, PTM

## Abstract

**Background:**

Gastrointestinal stromal tumor (GIST) is a common digestive tract tumor with high rate of metastasis and recurrence. Currently, we understand the genome, transcriptome and proteome in GIST. However, posttranscriptional modification features in GIST remain unclear. In the present study, we aimed to construct a complete profile of acetylome in GIST.

**Methods:**

Five common protein modifications, including acetylation, succinylation, crotonylation, 2-hydroxyisobutyrylation, and malonylation were tested among GIST subgroups and significantly differentially- expressed lysine acetylation was found. The acetylated peptides labeled with Tandem Mass Tag (TMT)under high sensitive mass spectrometry, and some proteins with acetylation sites were identified. Subsequently, these proteins and peptides were classified into high/moderate (H/M) risk and low (L) risk groups according to the modified NIH classification standard. Furthermore, cell components, molecular function, biological processes, KEGG pathways and protein interaction networks were analyzed.

**Results:**

A total of 2904 acetylation sites from 1319 proteins were identified, of which quantitative information of 2548 sites from 1169 proteins was obtained. Finally, the differentially-expressed lysine acetylation sites were assessed and we found that 42 acetylated sites of 38 proteins were upregulated in the H/M risk group compared with the L risk group, while 48 acetylated sites of 44 proteins were downregulated, of which Ki67 K1063Ac and FCHSD2 K24Ac were the two acetylated proteins that were most changed.

**Conclusions:**

Our novel findings provide further understanding of acetylome in GIST and might demonstrate the possibility in the acetylation targeted diagnosis and therapy of GIST.

**Supplementary Information:**

The online version contains supplementary material available at 10.1186/s12014-021-09322-0.

## Background

Gastrointestinal stromal tumors (GISTs) are the most common type of mesenchymal tumors of the gastrointestinal tract and originate from interstitial cells of Cajal [1]. GISTS were originally known as leiomyomas or epithelial leiomyosarcoma because GISTs mostly occur in the muscular layer of hollow organs [1, 2]. Until 1983, Mazur and Clark [3] used the name gastrointestinal stromal tumors, including all interstitial-derived tumors, as well as non-epithelial tumors of varying degrees of differentiation, such as leiomyoma and Schwannoma. GISTs mostly occur in the elderly, usually under the mucosa, but also in other parts of the gastrointestinal tract and are common in the stomach [4]. Deepening our understanding of the research has led to further understanding of the pathology and treatment of this disease. At present, most GISTs have shown to bear c-kit proto-oncogene mutations, and express CD117 and CD34 [5, 6]. KIT mutations are most common, and using molecular analysis, several mutation locations have been identified, including exons 9, 11, 13, and 17 [7]. Up to now, there is no authoritative and accurate epidemiological data on GISTs in China. Relevant medical research in the United States of America shows that GISTs have an annual incidence of 11.0 to 19.6 individuals/1 million individuals [8, 9].

Imatinib mesylate, a small molecule inhibitor of KIT and PDGFRA, yields long-lasting responses in most patients, with a median survival of almost 5 years [10]. However, 20% of patients show primary resistance to imatinib, and most responding patients eventually develop secondary resistance and disease progression [11, 12]. The response rate for second-line treatment with sunitinib was 7%, however, patients progressed after an average of 6 months, and the results at the time of progression were frustrating [13]. Consequently, there is an unmet need for alternative treatment strategies to treat GISTs.

Evidence has shown that protein post-translational modification (PTM) might contribute as a complement of therapeutic target for malignant tumors [14, 15]. Initially and during progression, epigenetic alteration, which leads to modified gene expression was involved in GISTs, [16–18]. The type of PTM that plays a central role in GISTs is still unknown. Moreover, no information is available about the target of protein PTM in the treatment of GISTs. Therefore, it is necessary to identify the main PTM and the differentially-expressed PTM proteins. In this study, we discovered lysine acetylation as the major changed PTM in GISTs, and used integrated approach involving tandem mass tag (TMT) labeling and mass spectrometry-based quantitative proteomics to quantify dynamic changes of protein acetylation including histone or non-histone proteins among GISTs samples of different grades.

## Methods

### Human tissue specimens

After the patient was pathologically diagnosed with GIST, human tissue specimens were collected perioperatively. Tissue samples were obtained in less than 30 min of ischemia time, then quickly frozen in liquid nitrogen until use. At each risk level (high risk, moderate risk and low risk groups), we selected three patients’ clinical tissue samples for subsequent mass spectrometry testing. Written informed consent was provided by all patients before sample collection. This study was approved by the local Research Ethics Committee of Peking University People’s Hospital (Beijing, China).

### Protein extraction

Samples were taken out of the − 80 °C, and about 1 g tissue sample was weighed into a mortar pre-cooled with liquid nitrogen, then grinded in liquid nitrogen into cell powder, and transferred to a 5-mL centrifuge tube. Next, four volumes of lysis buffer (8 M urea, 1% Protease Inhibitor Cocktail) was added to the cell powder, followed by three sonications on ice using a high intensity ultrasonic processor (Scientz, Ningbo, China). For PTM experiments, inhibitors were added to the lysis buffer, including 3 μM TSA (Trichostatin A) and 50 mM NAM (Nicotinamide) for acetylation. The remaining debris was removed by centrifugation at 12,000 g at 4 °C for 10 min. Finally, the supernatant was collected and the protein concentration was determined using BCA kit according to the manufacturer’s instructions.

### Western blot assay

Total proteins were extracted from 9 GISTs tissue samples (3 of each grade) lysis buffer. Lysates were denatured with SDS sample buffer at 95 °C for 5 min, and proteins were separated in 8–12% polyacrylamide gels, then transferred to nitrocellulose blotting (NC) membranes (Millipore, Massachusetts, United States). Membranes were blocked with 5% non-fat milk powder in TBST buffer for an hour at home temperature, and incubated with primary antibodies overnight at 4 °C. Anti-acetyllysine, anti-succinyllysine, anti-crotonyllysine, anti-2-hydroxyisobutyryllysine, and anti-malonyllysine antibodies were used as the primary antibodies. All of the primary antibodies were purchased from PTM Biolabs lnc. (Hangzhou, China). Then, membranes were washed with TBST buffer and incubated with the second antibody Goat anti-Mouse IgG (Pierce™, Thermo Scientific, USA) at room temperature for 45 min.

### Trypsin digestion

For digestion, the protein solution was reduced with 5 mM dithiothreitol for 30 min at 56 °C, and alkylated with 11 mM iodoacetamide for 15 min at room temperature in the dark. Then, the protein sample was diluted by adding 100 mM TEAB to a urea concentration of less than 2 M. Finally, for the first digestion, trypsin was added at a 1:50 trypsin-to-protein mass ratio overnight and 1:100 trypsin-to-protein mass ratio was added for a second 4 h-digestion.

### TMT labeling

After trypsin digestion, peptides were desalted by a Strata X C18 SPE column (Phenomenex, California, United States) and vacuum-dried. Peptides were reconstituted in 0.5 M TEAB and processed according to the manufacturer’s guidelines provided with the TMT kit. In brief, one unit of TMT reagent was thawed and reconstituted in acetonitrile. Subsequently, peptide mixtures were incubated for 2 h at room temperature and pooled, desalted, and dried by vacuum centrifugation.

### Affinity enrichment

Pan acetylation antibody-based PTM enrichment: To enrich acetylated peptides, tryptic peptides dissolved in NETN buffer (100 mM NaCl, 1 mM EDTA, 50 mM Tris–HCl, 0.5% NP-40, pH 8.0) were incubated with pre-washed antibody beads (Lot number 001, PTM Bio) at 4 °C overnight with gentle shaking. Then, the beads were washed four times. Bound peptides were eluted from the beads with 0.1% trifluoroacetic acid. Finally, the eluted fractions were combined and vacuum-dried. For LC–MS/MS analysis, the resulting peptides were desalted with C18 ZipTips (Millipore, Massachusetts, United States) according to the manufacturer’s instructions.

### LC–MS/MS analysis

The tryptic peptides were dissolved in 0.1% formic acid (solvent A), and directly loaded onto a home-made reversed-phase analytical column (15-cm length, 75 μm i.d.). The gradient was comprised of an increase from 6 to 23% solvent B (0.1% formic acid in 98% acetonitrile) over 26 min, 23% to 35% in 8 min, and increased to 80% in 3 min, then it was set to 80% for the last 3 min, all at a constant flow rate of 400 nL/min on an EASY-nLC 1000 UPLC system. The peptides were subjected to NSI source followed by tandem mass spectrometry (MS/MS) in Q ExactiveTM Plus (Thermo Fisher Scientific, Massachusetts, United States) coupled to the UPLC. The electrospray voltage applied was 2.0 kV. The m/z scan range was 350 to 1800 for full scan, and intact peptides were detected in the Orbitrap at a resolution of 70,000. Next, peptides were selected for MS/MS using ab NCE setting of 28 and fragments were detected in the Orbitrap at a resolution of 17,500. A data-dependent procedure that alternated between one MS scan was followed by 20 MS/MS scans with 15.0 s dynamic exclusion. Automatic gain control (AGC) was set at 5E4, and the fixed first mass was set as 100 m/z.

### Database search

Resulting MS/MS data were processed using a Maxquant search engine (v.1.5.2.8). Search parameter settings were as follows: the database used was human_swissprot_9606 (SwissProt Human, 20,422 sequences). Tandem mass spectra were searched against the human uniprot database concatenated with the reverse decoy database. Trypsin/P was specified as the cleavage enzyme allowing up to 4 missing cleavages. The mass tolerance for precursor ions was set to 20 ppm in the First search, 5 ppm in the Main search, and the mass tolerance for fragment ions was set to 0.02 Da. Carbamidomethyl on Cys was specified as fixed modification and acetylation modification and oxidation on Met were specified as variable modifications. The false discovery rate (FDR) of spectrum and protein were all set as 1% and the minimum score for modified peptides was set to > 40.

### GO annotation

The Gene Ontology (GO) annotation proteome was derived from the UniProt-GOA database ( http://www.ebi.ac.uk/GOA/). Firstly, the identified protein ID was converted to the UniProt ID, then mapped to GO IDs by protein ID. If identified proteins were not annotated by the UniProt-GOA database, the InterProScan software was used to annotate the protein’s GO functionally based on the protein sequence alignment method. Then proteins were classified by GO annotation based on three categories: biological process, cellular component, and molecular function.

### Domain annotation

The functional description of the identified proteins domain was annotated by InterProScan (a sequence analysis application) based on the protein sequence alignment method, and the InterPro domain database was used. InterPro (http://www.ebi.ac.uk/interpro/) is a database that integrates diverse information about protein families, domains, and functional sites, and makes it freely available to the public via Web-based interfaces and services. Central to the database are diagnostic models, also known as signatures, against which protein sequences can be searched to determine their potential function. InterPro has utility in the large-scale analysis of whole genomes and meta-genomes, as well as in characterizing individual protein sequences.

### KEGG pathway annotation

Kyoto Encyclopedia of Genes and Genomes (KEGG) Pathways mainly include: Metabolism, Genetic Information Processing, Environmental Information Processing, Cellular Processes, Rat Diseases, and Drug development. The KEGG database was used to annotate the protein pathway. First, the KEGG online service tool KAAS was used to annotate the protein’s KEGG database description. Then, the annotation result was mapped on the KEGG pathway database using the KEGG online service tool KEGG mapper.

### Subcellular localization

There, we used wolfpsort a subcellular localization predication software to predict subcellular localization. Wolfpsort is an updated version of PSORT/PSORT II and is used for the prediction of eukaryotic sequences. For protokaryon species, the subcellular localization prediction software CELLO was used.

### Enrichment-based clustering

For further hierarchical clustering based on differentially-modified protein functional classification (such as: GO, Domain, Pathway, Complex), we first collated all categories obtained after enrichment along with their P values, then filtered for categories which were at least enriched in one of the clusters with a P value < 0.05. The filtered P value matrix was transformed by the function x =  − log10 (P value). Finally, these x values were z-transformed for each functional category. Z scores were then clustered by one-way hierarchical clustering (Euclidean distance, average linkage clustering) in Genesis.

### Protein–protein interaction network

For protein–protein interactions, all differentially- expressed modified protein database accession or sequence were searched against STRING database version 10.1. Only interactions between proteins belonging to the searched data set were selected, thereby excluding external candidates. STRING defines a metric called “confidence score” to define the interaction confidence; all interactions that had a confidence score ≥ 0.7 (high confidence) were obtained.

## Results

### Acetylation is a key post-modification in GISTs

**Fig. 1:**
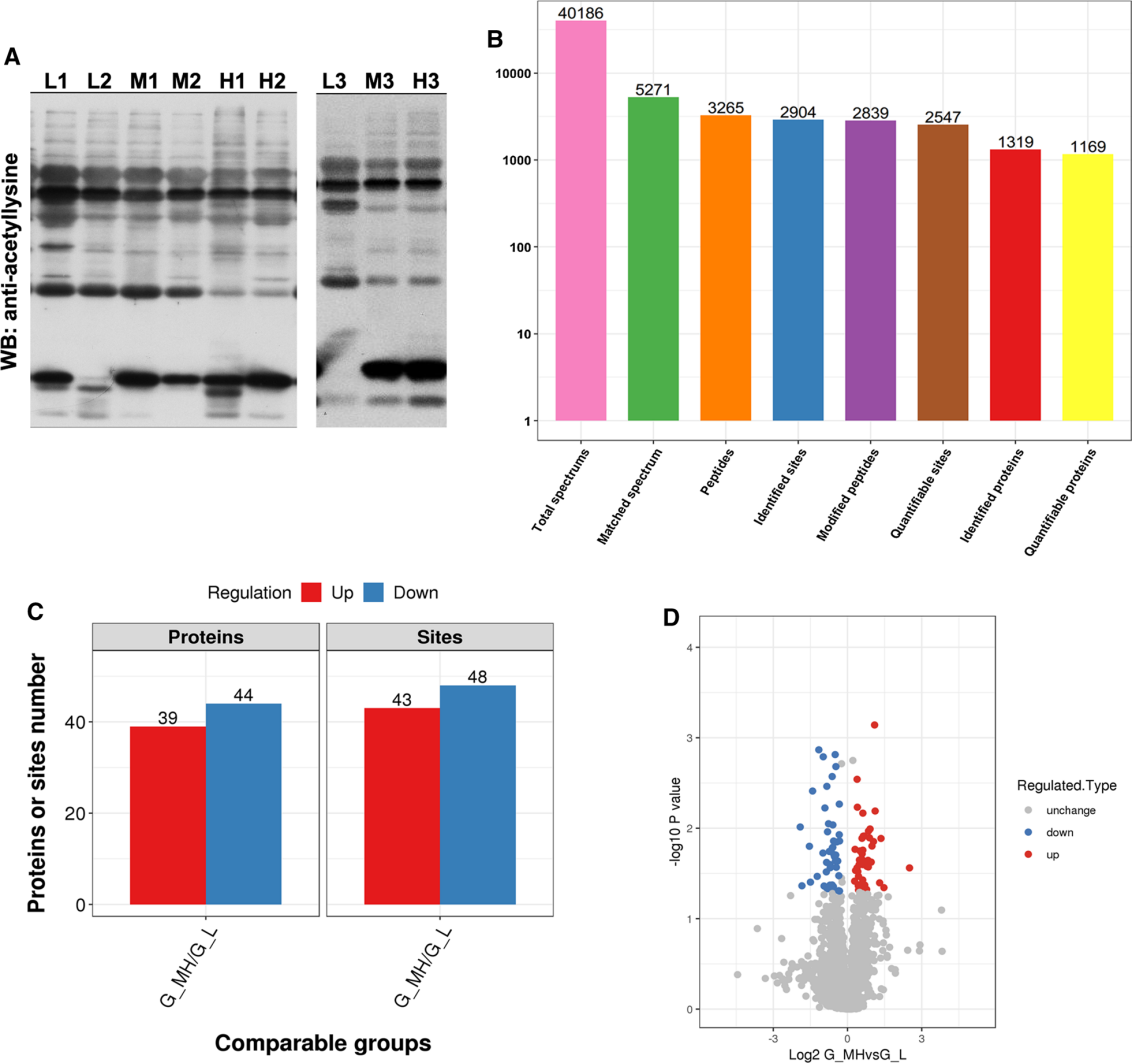
Distribution of identified proteins. **A** Western blotting with pan anti-acetyllysine antibody in 9 GIST tissue samples. **B** Quantification overview of acetylated sites and proteins from the mass spectrum. **C** Differentially expressed acetylated sites and proteins between GIST moderate/high risk group and low risk group integrated all sample data. **D** Volcano map of differentially expressed acetylated sites. Red points represent upregulation sites while blue points indicate downregulation sites

Western blot results showed that under the same conditions, the number of bands displayed by the pan-acetylated antibody was higher, and the acetylated bands were more obvious under the same exposure time compared with other modifications. Moreover, acetylation was found to be changed most in the five PTM above between high risk and low/moderate risk (Fig. 1A, Additional file 1: Figure S1). The tissue samples are divided into different risk levels according to the modified NIH classification standard. Thus, acetylation was selected for further studies.

### Quality control validation of MS data

As shown in Figure S2A, most peptides are distributed between 7 and 20 amino acids, which conforms to the general rule of trypsin-based enzymolysis and HCD fragmentation. The distribution of peptide lengths identified by mass spectrometry met the quality control requirements. The first-order mass error of most of the spectra was within 10 ppm, which meets the high-precision characteristics of the mass spectrum. This showed that the mass accuracy of the mass spectrometer was in the normal range, and that the qualitative and quantitative analysis of proteins will not be affected due to the large mass deviation. The score of the spectrally matched peptides (characterizing the credibility of the peptide identification) negatively correlated with the distribution of mass deviations. The higher the s core, the smaller the quality deviation (Additional file 1: Figure S2B).

### Quantification overview of acetylated sites and proteins

Raw MS files were processed within the MaxQuant environment (version 1.5.2.8) using the integrated Andromeda search engine with FDR < 0.01 at the protein, peptide, and modification level. In the study, a total of 40,186 MS/MS spectrum were obtained by mass spectrometry. Analysis result shown that 5271(13.1%) spectrum matched with 3265 peptide, among which 2839 was acetyalted peptides. A total of 2904 acetylation sites on 1319 were identified, and 2547 sites on1169 proteins were quantifiable (Fig. 1B). To thoroughly understand the modified proteins identified and quantified in the data, detailed annotations on the functions and characteristics of these proteins were made from the aspects of GO, protein domain, KEGG pathway, and localization of subcellular structures.

### Protein annotation

Our first step was to calculate the difference between the modification sites in the comparison group. When the P-value was less than 0.05, the change in the amount of differential modification exceeded 1.2 as the change threshold that was significantly increased, and less than 0.833 as the change threshold that was significantly decreased. Our results showed that compared to low-risk group, in the medium and high risk group, 44 of acetylated proteins were downregulated and 39 were upregulated while 48 acetylated sites were downregulated and 43 acetylated sites were upregulated (Table 1, Fig. 1C, D).

**Table 1:** Differentially expressed acetylation sites obtained in 9 GIST samples

Protein accession	Protein description	Position	G_MH/G_L Ratio	Regulated Type	G_MH/G_L P value
A0A024RBG1	Diphosphoinositol polyphosphate phosphohydrolase NUDT4B OS=Homo sapiens OX=9606 GN=NUDT4B PE=3 SV=1	134	0.672	Down	0.044143
O00193	Small acidic protein OS=Homo sapiens OX=9606 GN=SMAP PE=1 SV=1	13	2.136	Up	0.00072261
O60563	Cyclin-T1 OS=Homo sapiens OX=9606 GN=CCNT1 PE=1 SV=1	638	1.707	Up	0.047559
O60934	Nibrin OS=Homo sapiens OX=9606 GN=NBN PE=1 SV=1	544	1.308	Up	0.027417
O75874	Isocitrate dehydrogenase [NADP] cytoplasmic OS=Homo sapiens OX=9606 GN=IDH1 PE=1 SV=2	87	1.319	Up	0.0058635
O94868	F-BAR and double SH3 domains protein 2 OS=Homo sapiens OX=9606 GN=FCHSD2 PE=1 SV=3	24	0.266	Down	0.0096962
P00450	Ceruloplasmin OS=Homo sapiens OX=9606 GN=CP PE=1 SV=1	712	0.571	Down	0.046757
P00738	Haptoglobin OS=Homo sapiens OX=9606 GN=HP PE=1 SV=1	291	0.448	Down	0.00135938
P00738	Haptoglobin OS=Homo sapiens OX=9606 GN=HP PE=1 SV=1	321	0.653	Down	0.0165435
P00966	Argininosuccinate synthase OS=Homo sapiens OX=9606 GN=ASS1 PE=1 SV=2	340	0.612	Down	0.025202
P01024	Complement C3 OS=Homo sapiens OX=9606 GN=C3 PE=1 SV=2	1360	0.687	Down	0.048143
P02647	Apolipoprotein A-I OS=Homo sapiens OX=9606 GN=APOA1 PE=1 SV=1	219	0.557	Down	0.023881
P02787	Serotransferrin OS=Homo sapiens OX=9606 GN=TF PE=1 SV=3	618	0.662	Down	0.0162807
P04406	Glyceraldehyde-3-phosphate dehydrogenase OS=Homo sapiens OX=9606 GN=GAPDH PE=1 SV=3	117	2.461	Up	0.040164
P06753	Tropomyosin alpha-3 chain OS=Homo sapiens OX=9606 GN=TPM3 PE=1 SV=2	153	1.538	Up	0.006816
P07585	Decorin OS=Homo sapiens OX=9606 GN=DCN PE=1 SV=1	164	0.678	Down	0.0138641
P08133	Annexin A6 OS=Homo sapiens OX=9606 GN=ANXA6 PE=1 SV=3	306	1.351	Up	0.043101
P08133	Annexin A6 OS=Homo sapiens OX=9606 GN=ANXA6 PE=1 SV=3	9	1.62	Up	0.042521
P08237	ATP-dependent 6-phosphofructokinase, muscle type OS=Homo sapiens OX=9606 GN=PFKM PE=1 SV=2	476	1.469	Up	0.043421
P08571	Monocyte differentiation antigen CD14 OS=Homo sapiens OX=9606 GN=CD14 PE=1 SV=2	119	0.553	Down	0.030401
P08670	Vimentin OS=Homo sapiens OX=9606 GN=VIM PE=1 SV=4	373	0.708	Down	0.00153584
P08708	40S ribosomal protein S17 OS=Homo sapiens OX=9606 GN=RPS17 PE=1 SV=2	44	1.373	Up	0.042363
P10412	Histone H1.4 OS=Homo sapiens OX=9606 GN=HIST1H1E PE=1 SV=2	75	0.784	Down	0.033478
P11021	Endoplasmic reticulum chaperone BiP OS=Homo sapiens OX=9606 GN=HSPA5 PE=1 SV=2	213	0.8	Down	0.0138592
P12429	Annexin A3 OS=Homo sapiens OX=9606 GN=ANXA3 PE=1 SV=3	58	1.505	Up	0.0192233
P13010	X-ray repair cross-complementing protein 5 OS=Homo sapiens OX=9606 GN=XRCC5 PE=1 SV=3	565	1.942	Up	0.023699
P13987	CD59 glycoprotein OS=Homo sapiens OX=9606 GN=CD59 PE=1 SV=1	63	0.612	Down	0.042604
P17844	Probable ATP-dependent RNA helicase DDX5 OS=Homo sapiens OX=9606 GN=DDX5 PE=1 SV=1	197	1.222	Up	0.038495
P17844	Probable ATP-dependent RNA helicase DDX5 OS=Homo sapiens OX=9606 GN=DDX5 PE=1 SV=1	33	1.361	Up	0.034019
P17858	ATP-dependent 6-phosphofructokinase, liver type OS=Homo sapiens OX=9606 GN=PFKL PE=1 SV=6	469	1.335	Up	0.030163
P19338	Nucleolin OS=Homo sapiens OX=9606 GN=NCL PE=1 SV=3	96	1.722	Up	0.026438
P19827	Inter-alpha-trypsin inhibitor heavy chain H1 OS=Homo sapiens OX=9606 GN=ITIH1 PE=1 SV=3	312	0.588	Down	0.0089201
P19827	Inter-alpha-trypsin inhibitor heavy chain H1 OS=Homo sapiens OX=9606 GN=ITIH1 PE=1 SV=3	222	0.507	Down	0.00162407
P20700	Lamin-B1 OS=Homo sapiens OX=9606 GN=LMNB1 PE=1 SV=2	156	0.78	Down	0.049082
P24821	Tenascin OS=Homo sapiens OX=9606 GN=TNC PE=1 SV=3	1011	0.354	Down	0.039499
P27816	Microtubule-associated protein 4 OS=Homo sapiens OX=9606 GN=MAP4 PE=1 SV=3	769	0.617	Down	0.027344
P32119	Peroxiredoxin-2 OS=Homo sapiens OX=9606 GN=PRDX2 PE=1 SV=5	26	1.502	Up	0.022156
P35241	Radixin OS=Homo sapiens OX=9606 GN=RDX PE=1 SV=1	400	0.519	Down	0.043557
P35579	Myosin-9 OS=Homo sapiens OX=9606 GN=MYH9 PE=1 SV=4	972	1.236	Up	0.0171213
P35579	Myosin-9 OS=Homo sapiens OX=9606 GN=MYH9 PE=1 SV=4	1775	2.07	Up	0.0140371
P35579	Myosin-9 OS=Homo sapiens OX=9606 GN=MYH9 PE=1 SV=4	1459	1.309	Up	0.0028802
P36578	60S ribosomal protein L4 OS=Homo sapiens OX=9606 GN=RPL4 PE=1 SV=5	239	1.423	Up	0.0178187
P41218	Myeloid cell nuclear differentiation antigen OS=Homo sapiens OX=9606 GN=MNDA PE=1 SV=1	55	0.663	Down	0.0091989
P45974	Ubiquitin carboxyl-terminal hydrolase 5 OS=Homo sapiens OX=9606 GN=USP5 PE=1 SV=2	357	1.384	Up	0.025318
P46013	Proliferation marker protein Ki-67 OS=Homo sapiens OX=9606 GN=MKI67 PE=1 SV=2	1063	5.681	Up	0.027464
P47756	F-actin-capping protein subunit beta OS=Homo sapiens OX=9606 GN=CAPZB PE=1 SV=4	235	1.399	Up	0.022458
P47895	Aldehyde dehydrogenase family 1 member A3 OS=Homo sapiens OX=9606 GN=ALDH1A3 PE=1 SV=2	373	2.773	Up	0.0454
P48163	NADP-dependent malic enzyme OS=Homo sapiens OX=9606 GN=ME1 PE=1 SV=1	60	0.731	Down	0.02706
P51659	Peroxisomal multifunctional enzyme type 2 OS=Homo sapiens OX=9606 GN=HSD17B4 PE=1 SV=3	139	1.529	Up	0.025245
P53396	ATP-citrate synthase OS=Homo sapiens OX=9606 GN=ACLY PE=1 SV=3	978	1.791	Up	0.026841
P57740	Nuclear pore complex protein Nup107 OS=Homo sapiens OX=9606 GN=NUP107 PE=1 SV=1	85	1.363	Up	0.048177
P61086	Ubiquitin-conjugating enzyme E2 K OS=Homo sapiens OX=9606 GN=UBE2K PE=1 SV=3	14	1.529	Up	0.037338
P63010	AP-2 complex subunit beta OS=Homo sapiens OX=9606 GN=AP2B1 PE=1 SV=1	318	0.76	Down	0.023057
P80511	Protein S100-A12 OS=Homo sapiens OX=9606 GN=S100A12 PE=1 SV=2	91	1.881	Up	0.0102004
Q01082	Spectrin beta chain, non-erythrocytic 1 OS=Homo sapiens OX=9606 GN=SPTBN1 PE=1 SV=2	2344	0.722	Down	0.0020834
Q03252	Lamin-B2 OS=Homo sapiens OX=9606 GN=LMNB2 PE=1 SV=4	298	0.651	Down	0.042461
Q05682	Caldesmon OS=Homo sapiens OX=9606 GN=CALD1 PE=1 SV=3	618	1.986	Up	0.0157559
Q07065	Cytoskeleton-associated protein 4 OS=Homo sapiens OX=9606 GN=CKAP4 PE=1 SV=2	388	0.612	Down	0.045483
Q07666	KH domain-containing, RNA-binding, signal transduction-associated protein 1 OS=Homo sapiens OX=9606 GN=KHDRBS1 PE=1 SV=1	152	1.442	Up	0.022284
Q13813	Spectrin alpha chain, non-erythrocytic 1 OS=Homo sapiens OX=9606 GN=SPTAN1 PE=1 SV=3	1486	0.684	Down	0.0195798
Q15075	Early endosome antigen 1 OS=Homo sapiens OX=9606 GN=EEA1 PE=1 SV=2	791	0.695	Down	0.020596
Q15582	Transforming growth factor-beta-induced protein ig-h3 OS=Homo sapiens OX=9606 GN=TGFBI PE=1 SV=1	60	0.428	Down	0.03408
Q15582	Transforming growth factor-beta-induced protein ig-h3 OS=Homo sapiens OX=9606 GN=TGFBI PE=1 SV=1	53	0.799	Down	0.049475
Q16563	Synaptophysin-like protein 1 OS=Homo sapiens OX=9606 GN=SYPL1 PE=1 SV=1	194	0.561	Down	0.0034449
Q5QNW6	Histone H2B type 2-F OS=Homo sapiens OX=9606 GN=HIST2H2BF PE=1 SV=3	24	1.536	Up	0.0174835
Q5VTE0	Putative elongation factor 1-alpha-like 3 OS=Homo sapiens OX=9606 GN=EEF1A1P5 PE=5 SV=1	41	0.793	Down	0.0117988
Q6NZI2	Caveolae-associated protein 1 OS=Homo sapiens OX=9606 GN=CAVIN1 PE=1 SV=1	326	0.376	Down	0.0038804
Q86T29	Zinc finger protein 605 OS=Homo sapiens OX=9606 GN=ZNF605 PE=2 SV=1	118	0.719	Down	0.0197837
Q96EV2	RNA-binding protein 33 OS=Homo sapiens OX=9606 GN=RBM33 PE=1 SV=3	756	1.775	Up	0.022658
Q96G03	Phosphoglucomutase-2 OS=Homo sapiens OX=9606 GN=PGM2 PE=1 SV=4	36	1.255	Up	0.029339
Q96IX5	ATP synthase membrane subunit DAPIT, mitochondrial OS=Homo sapiens OX=9606 GN=ATP5MD PE=1 SV=1	16	0.622	Down	0.046917
Q96JC9	ELL-associated factor 1 OS=Homo sapiens OX=9606 GN=EAF1 PE=1 SV=1	150	1.501	Up	0.0129383
Q96L91	E1A-binding protein p400 OS=Homo sapiens OX=9606 GN=EP400 PE=1 SV=4	1160	0.609	Down	0.043665
Q96TA1	Niban-like protein 1 OS=Homo sapiens OX=9606 GN=FAM129B PE=1 SV=3	479	0.344	Down	0.0158443
Q99612	Krueppel-like factor 6 OS=Homo sapiens OX=9606 GN=KLF6 PE=1 SV=3	228	1.553	Up	0.045342
Q9BR76	Coronin-1B OS=Homo sapiens OX=9606 GN=CORO1B PE=1 SV=1	21	0.279	Down	0.043319
Q9BUL5	PHD finger protein 23 OS=Homo sapiens OX=9606 GN=PHF23 PE=1 SV=1	211	1.85	Up	0.0127642
Q9BWH2	FUN14 domain-containing protein 2 OS=Homo sapiens OX=9606 GN=FUNDC2 PE=1 SV=2	161	0.601	Down	0.0180819
Q9BWH2	FUN14 domain-containing protein 2 OS=Homo sapiens OX=9606 GN=FUNDC2 PE=1 SV=2	150	0.572	Down	0.0109557
Q9H147	Deoxynucleotidyltransferase terminal-interacting protein 1 OS=Homo sapiens OX=9606 GN=DNTTIP1 PE=1 SV=2	147	1.557	Up	0.0122376
Q9H223	EH domain-containing protein 4 OS=Homo sapiens OX=9606 GN=EHD4 PE=1 SV=1	378	0.795	Down	0.0054175
Q9P0V9	Septin-10 OS=Homo sapiens OX=9606 GN=SEPT10 PE=1 SV=2	377	0.531	Down	0.0059765
Q9UJZ1	Stomatin-like protein 2, mitochondrial OS=Homo sapiens OX=9606 GN=STOML2 PE=1 SV=1	140	0.551	Down	0.045145
Q9ULV4	Coronin-1C OS=Homo sapiens OX=9606 GN=CORO1C PE=1 SV=1	19	0.742	Down	0.0141771
Q9UQ35	Serine/arginine repetitive matrix protein 2 OS=Homo sapiens OX=9606 GN=SRRM2 PE=1 SV=2	2602	2.555	Up	0.012981
Q9Y2W1	Thyroid hormone receptor-associated protein 3 OS=Homo sapiens OX=9606 GN=THRAP3 PE=1 SV=2	420	0.503	Down	0.0188206
Q9Y2W2	WW domain-binding protein 11 OS=Homo sapiens OX=9606 GN=WBP11 PE=1 SV=1	13	1.594	Up	0.0122
Q9Y4G6	Talin-2 OS=Homo sapiens OX=9606 GN=TLN2 PE=1 SV=4	287	0.714	Down	0.023017
Q9Y5B6	PAX3- and PAX7-binding protein 1 OS=Homo sapiens OX=9606 GN=PAXBP1 PE=1 SV=2	278	1.802	Up	0.0107447
Q9Y6N5	Sulfide:quinone oxidoreductase, mitochondrial OS=Homo sapiens OX=9606 GN=SQOR PE=1 SV=1	372	0.651	Down	0.0026847
Q9Y6X0	SET-binding protein OS=Homo sapiens OX=9606 GN=SETBP1 PE=1 SV=3	747	2.17	Up	0.0064602

### Functional annotation of the Lys acetylome in GIST

We further explored the regulatory effect of lysine acetylation on cellular function, therefore, GO annotations were used which fell into three broad categories: Biological Process, Cellular Component, and Molecular Function. GO annotations can explain the biological role of proteins from different perspectives. We performed statistics on the distribution of proteins corresponding to differentially- modified sites in the secondary annotation of GO. In terms of the biological process, acetylated proteins are assigned to several groups. A total of 15% of acetylated proteins refer to cellular processes, 13% refers to biological regulation, and 12% refers to single-organism proteins. Moreover, downregulated or upregulated acetylated proteins in high and middle-risk tissues of GISTs mainly associate with cellular process (14% and 16%), biological regulation (12% and 13%), and single-organism processes (12% and 13%).

**Fig. 2:**
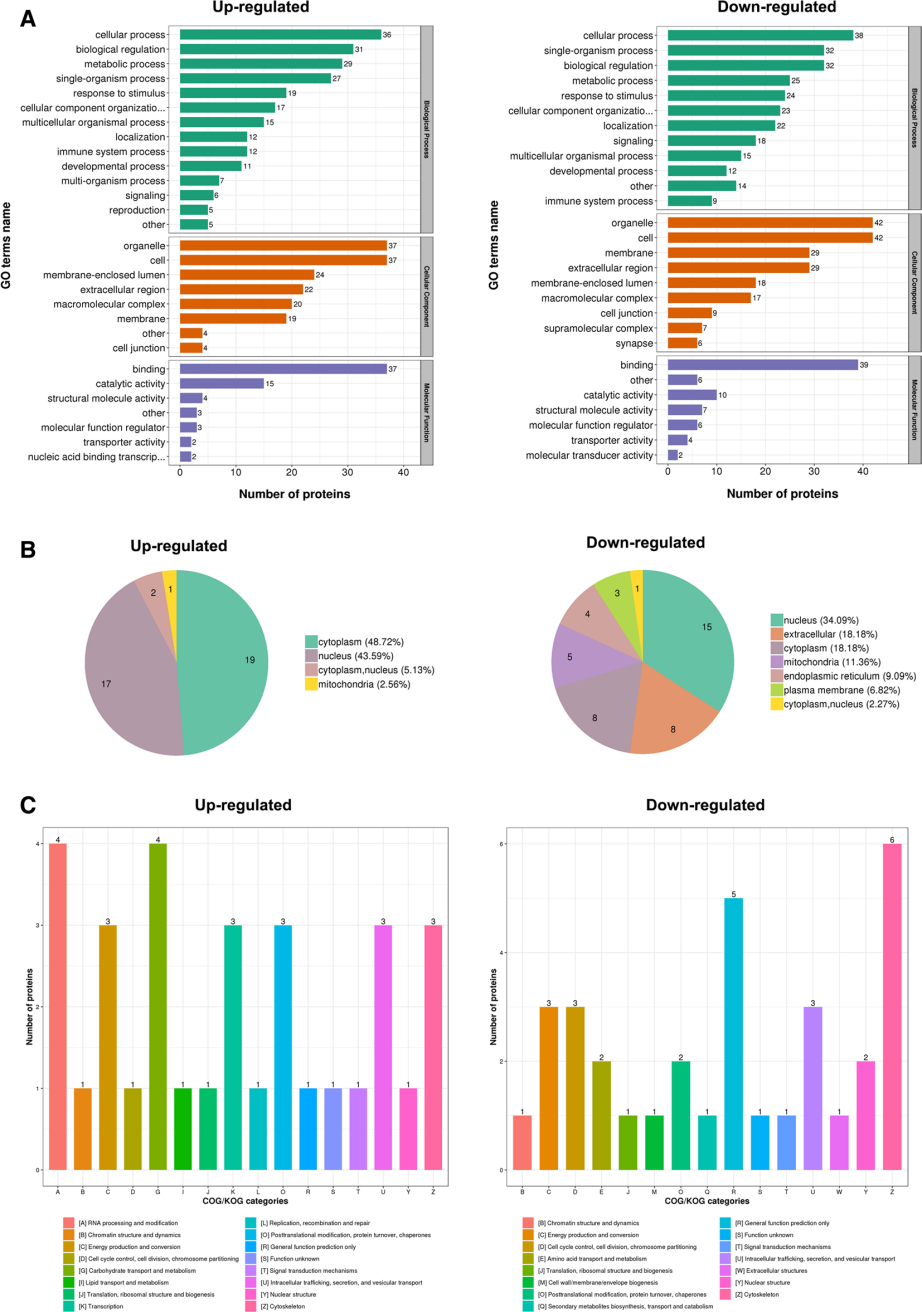
Functional characterization of identified acetylation proteins. **A** The classification of acetylated proteins in biological process, cellular component, and molecular function. **B** The located distribution of differential expressed acetylated proteins in the GIST cell. **C** COG / KOG functional classification statistics

Furthermore, the cellular components of acetylated proteins were also analyzed by the GO annotation method. Our results manifested that most acetylated proteins in GISTs are localized in the cell (22%), organelles (22%), extracellular region (14%), membrane (13%), and membrane enclosed lumen (11%). In addition, we also found that low-expression or overexpression of acetylated proteins in high and middle-risk tissues of GISTs compared to low-risk tissues were also primarily distributed in the cell (21% and 22%), organelles (21% and 22%), membrane (15% and 11%), extracellular region (15% and 13%), and membrane enclosed lumen (9% and 14%) (Fig. 2A).

Regarding molecular function analysis, it was shown that of the 140 acetylated proteins, 54% of substrates were associated with binding and 18% were related to catalytic activity. The remainder were involved in structural molecule activity 8%, molecular function regulator 7%, transporter activity 4%, nucleic and binding transcription factor activity 2%, etc. In high and middle-risk tissues of GIST, compared to that in low-risk tissues, under-expressed or overexpressed acetylated proteins were mainly involved in binding (53% and 56%) and catalytic activity (14% and 23%).

It was desirable to further analyze the precise location of different proteins expressed in a cell, therefore, we used specialized software for the difference in the modified protein was predicted subcellular structures and classification statistics, the results showed that the acetylated proteins in GIST were mainly localized in the nucleus (38.55%), cytoplasm (32.53%), extracellular region (9.64%), and mitochondria (7.23%). For further understanding the function of acetylated proteins in GIST, we evaluated the subcellular localization. The results showed that among all differentially- modified proteins compared with low-risk tissues of GIST, the lower-expression acetylated proteins were mainly distributed in high and middle-risk tissues. In addition, over-expression of acetylated proteins in high and middle-risk tissues of GIST, were found to be distributed in the cytoplasm (48.72%), nucleus (43.59%), cytoplasm, nucleus (5.13%), and mitochondria (2.56%) (Fig. 2B).

To achieve the purpose of exploring the differential protein category and their specific functions, we used database comparison analysis to perform Clusters of Orthologous Groups of proteins (COG / KOG) functional classification statistics of differentially- modified proteins. We found that acetylated proteins were widely involved in the cytoskeleton (14%), intracellular trafficking, secretion, and vesicular transport (10%), energy production and conversion (10%), general function prediction only (10%), and posttranslational modification, protein turnover, chaperones (8%) (Fig. 2C).

### Enrichment and clustering analysis of the Lys acetylation data sets

**Fig. 3:**
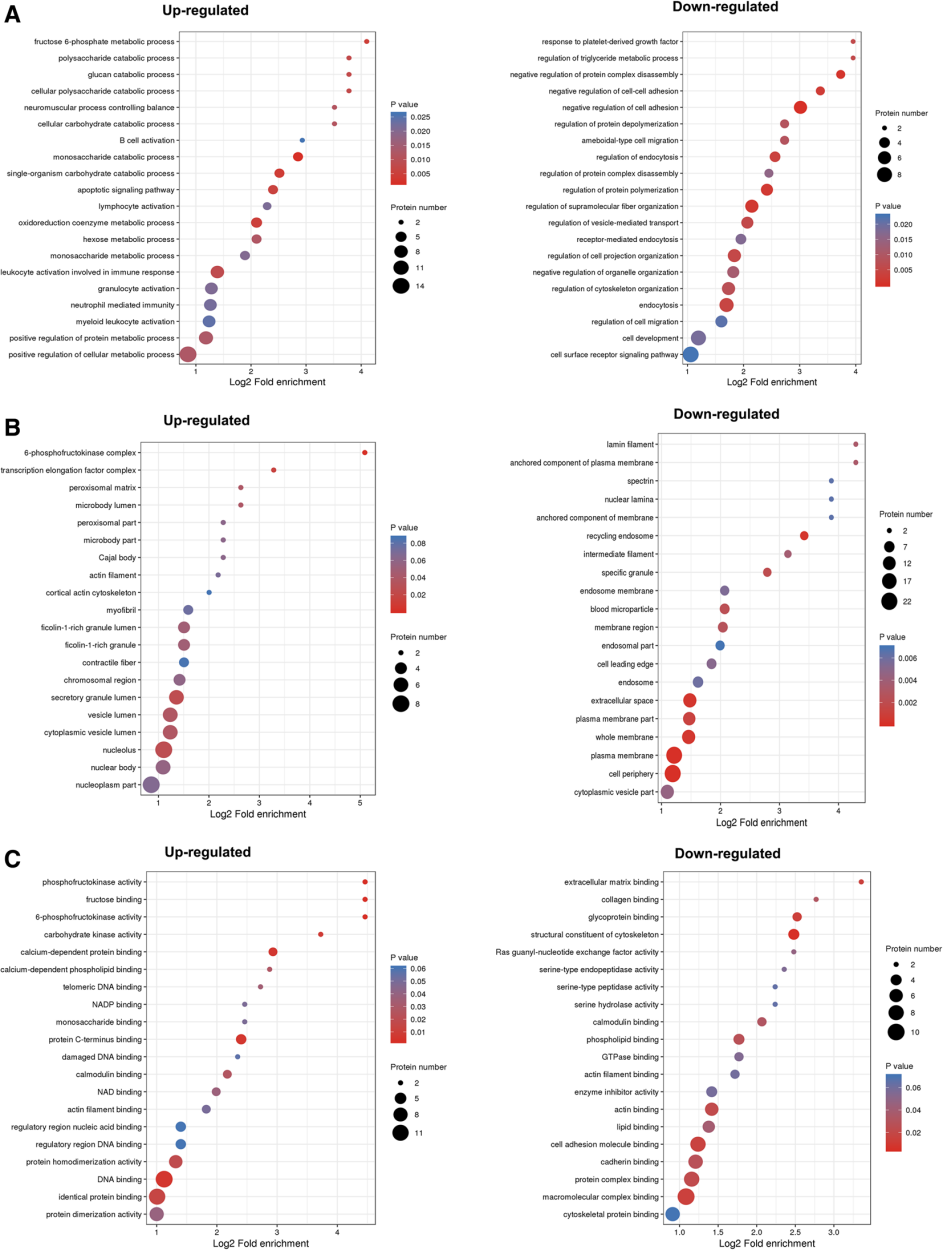
Enrichment clustering showed by bubble chart. Differential expressed lysine acetylated proteins between GIST moderate/high risk group and low risk group were classified by GO annotation based on the **A** biological process, **B** cellular component, and **C **molecular function

In our study, we focused on the repeated changes in acetylation in nine samples. To further clarify the cellular functions of these differential proteins in GIST at different risk levels, enrichment tests were performed on the data from three GO aspects: biological process, cell component, and molecular function. In the classification of biological processes, processes related to fructose 6-phosphate metabolic process and glucan catabolic process were significantly enriched in high and middle-risk tissues upregulated protein clusters, while responses to platelet-derived growth factor were significantly enriched in down-regulated proteins (Fig. 3A). Consistent with the above-mentioned observations, cell component analysis showed that acetylated proteins were mainly concentrated in the transcription elongation factor complex and secretory granule lumen of up-regulated proteins, while specific granules and recycling endosomes were enriched in down-regulated proteins (Fig. 3B). In addition, assessment of molecular function indicated that proteins involved in phospholipid binding and actin binding were enriched in down-regulated protein clusters, however, in up-regulated 6-phosphofructokinase activity and phosphofructokinase activity were increased (Fig. 3C).

The function of a protein depends heavily on the specific domains in the sequence. To assess the most regulated domains between different risk levels in GIST, protein domain enrichment analysis was performed. Consistent with our findings in biological processes and cellular components, we demonstrated that during the progressive severity of GIST, the protein domain of the NAD(P)-binding domain increased with increasing acetylation, while the PH domain-like increased most when the protein level decreased (Additional file 1: Figure S3A).

To identify cellular pathways that play an important role in GIST, we performed pathway clustering analysis from KEGG. Our data showed that primary bile acid biosynthesis and phenylalanine metabolism were the most significant pathways for enrichment in high and middle-risk GIST tissues with elevated levels of acetylation, and that the ferroptosis pathway that was down-regulated by acetylated proteins was the most abundant (Additional file 1: Figure S3B).

### Protein interaction networks of Lys acetylation proteome

**Fig. 4:**
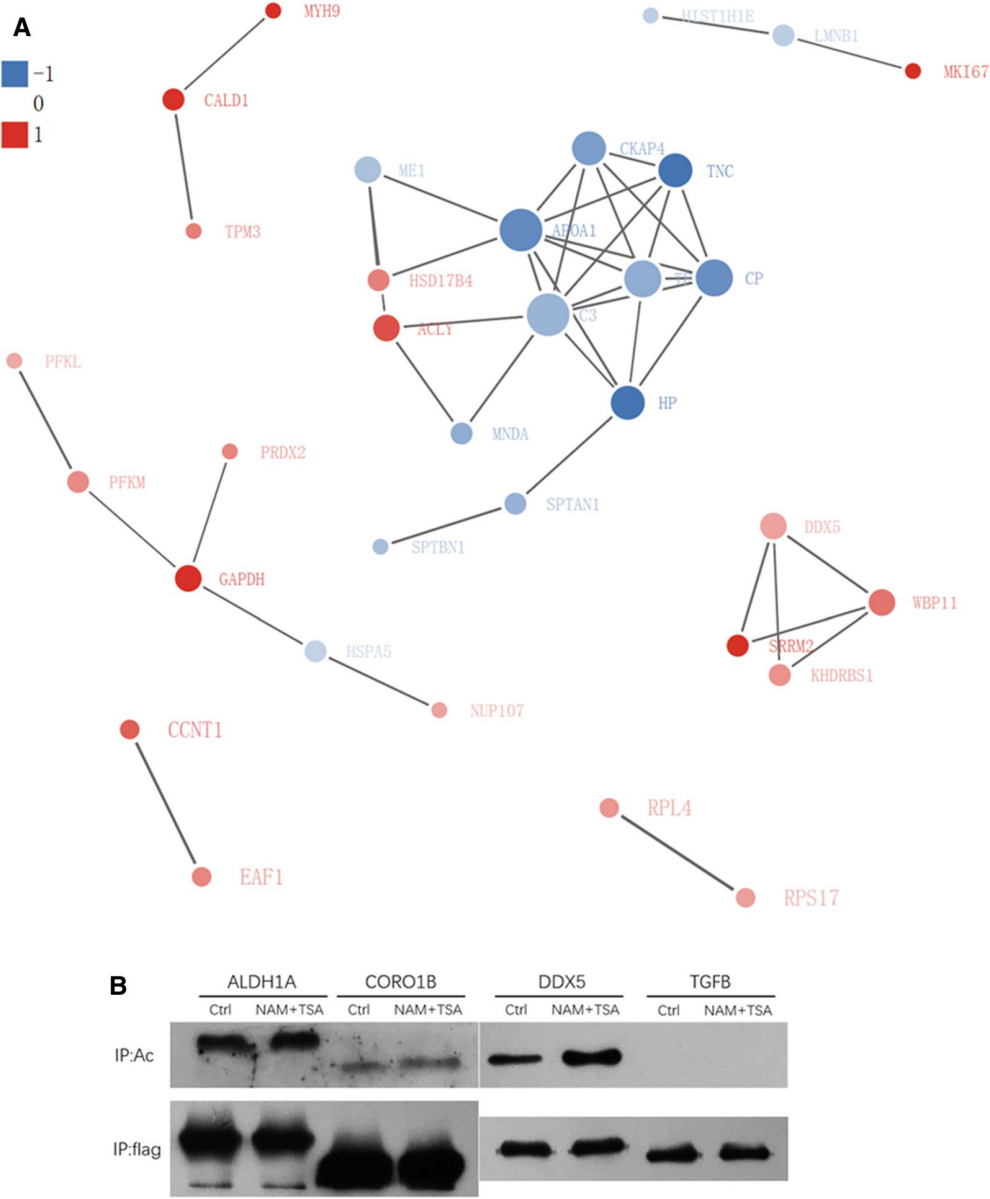
Representative protein–protein interaction network of Lys acetylation proteome. **A** Interaction networks were analyzed on the basis of the STRING database (v10.1). **B** Four differentially expressed interesting proteins were selected to be verified its acetylation in GISTs

The differentially- modified protein database numbers or protein sequences screened in different comparison groups were compared with the STRING protein network interaction database to extract differential protein interactions based on a confidence score > 0.7 (high confidence). Our data set provided insight into the interactions of acetylated proteins in GIST in tissues of varying risk. A typical example is shown in Fig. 4A. Using the MCODE tool, we identified some highly connected subnetworks of Lys-acetylated proteins, including ATP synthase, fat synthase, and cytoskeleton.

### Proteins are verified acetylated in GISTs

Four of our interesting differential proteins were selected to be verified whether they were in accordance with the results of mass spectrum analysis. As shown in Fig. 4b, ALDH1A, CORO1B, and DDX5 were able to be acetylated in GISTs, which demonstrated that the mass spectrum results were reliable.

## Discussion

GISTs are now stratified into four groups (very low, low, moderate, and high risk) according to the NIH consensus classification criteria based on tumor size and mitotic rates [19]. Different risk grades have different survivals, increasing from low to high risk [2]. Although GISTs were triggered by a c-kit mutation, some identical mutation types showed poor over survivals and distinct responses to imatinib [12, 20, 21]. Therefore, it is important to identify novel therapeutic targets for GISTs. In 2019, Liu et al. reported proteomics maps of human GISTs and indicated that patients with high PTPN1 expression had low risk of developing metastasis [22]. However, the global alteration of protein post-translational modifications in GISTs has been yet unknown.

Lysine acetylation was first discovered in 1964 as a PTM of histones. For the past few decades, Lys acetylation has been considered an important post-modification of histone and non-histone functions [23, 24]. Moreover, this modification has been demonstrated to affect the occurrence and progression of a variety of cancers [25–30]. However, studies on global acetylated protein have not been yet conducted on GISTs. Owing to technical limitations, the number of acetylated proteins far exceeds the expectations. In this study, we tested five common PTMs in GISTs and found that protein acetylation changed most. Next, we used quantitative acetylated proteomics to achieve a holistic view of the acetylated group in GIST at different risk levels.

Here, a quantitative study of acetylation-modified proteomics was performed. We identified 2904 acetylation modification sites on 1319 proteins, of which 2547 sites on 1169 proteins contained quantitative information. After analyzing all 9 GIST samples, comparing the high-risk group and the middle-risk group of the GIST with the low-risk group, we found that the acetylation modification levels at 43 sites were increased, and that the level of acetylation modification at 48 sites was down-regulated. P53 was the first nonhistone protein reported to have the potential of being modified by acetylation in humans [31]. In 2006, Kim et al. invented a new method to study global protein acetylation and reported about 400 lysine acetylation sites in almost 200 proteins [32]. In addition, Choudhary et al. [24] identified 3600 acetylation sites of 1750 acetylated proteins, thereby tremendously increasing the size of the acetylome. In recent years, nonhistone protein acetylation was thought to be tightly related to tumor progression. Our previous studies showed IDH1 K224 acetylation, regulated by SIRT2, affected cell metastasis in colorectal cancer [33]. However, the key acetylated proteins and acetylation sites in GISTs are not known. Our present findings have filled in that blank. In our results, of all differentially-expressed acetylated protiens, FCHSD2 K24Ac was the most significantly downregulated protein and Ki67 K1063Ac displayed the largest degree of upregulation in M/H groups. Thus, these two acetylated nonhistone proteins might serve as the key regulators contributing to risk of GISTs. This study is the first to demonstrate a correlation between acetylated proteins and different risk ratings for GIST. The expansion of the acetylated protein catalogue may be due to improved antibody specificity and recognition range as well as improved detection technology.

To further explore the characterization of these identified acetylated proteins, we utilized GO annotation to analyze potential functions of the acetylated proteins. Our results showed that the acetylated proteins of ectopic expression in high and middle-risk tissues of GIST were mainly distributed in cells, organelles, extracellular region, and membrane, and largely played a role in molecular binding and catalytic activity, thereby controlling cellular component and metabolic process. Moreover, through analysis of the KEGG pathway and protein-interaction networks, we discovered that carbon metabolism, biosynthesis of fat and the cytoskeleton were the most prominent pathways enriched in high and middle-risk tissues. In addition, ATP synthetase, cytoskeleton synthetase, and lecithin cholesterol acyltransferase might be crucial proteins during the progression of GIST. Understanding the structure of acetylated proteins may greatly improve our understanding of the role of acetylated proteins in the different risks of GIST.

It must be mentioned that the sample set for proteomic analysis in this study was relatively small, which might affect the quantification accuracy of proteins and make the results less robust. We look forward to studies with a larger sample cohort of GISTs, providing more and more inspiring discoveries.

## Conclusions

Using a quantitative proteomics research strategy of TMT labeling and acetylation modification enrichment technology and HPLC fractionation, in this study a complete atlas of acetylome in GIST was conducted. In total, 2904 acetylation sites from 1319 proteins, of which 2548 sites from 1169 proteins with quantitative information were identified. Finally, we assessed the differentially-expressed lysine acetylation sites and identified 42 acetylated sites of 38 proteins that were upregulated in the H/M risk group compared with the L risk group, while 48 acetylated sites of 44 proteins were downregulated, of which Ki67 K1063Ac and FCHSD2 K24Ac were the two acetylated proteins that were most changed. The findings of this study provide further understanding of the acetylome in GIST and might demonstrate probability in acetylation- targeted diagnosis and therapy of GIST.

The function of these proteins associated with different risk grades of GIST merits extensive investigation in future studies. Furthermore, this study provides many interesting potential proteins with acetylation sites for future studies.

## Supplementary Information

**Additional file 1: Figure S1.** Western blotting with pan anti-succinyllysine, anti-crotonyllysine, anti-2-hydroxyisobutyryllysine, and anti-malonyllysine antibodies in 9 GIST tissue samples. **Figure S2.** Quality control validation of MS data. The distribution of peptide lengths identified by mass spectrometry met quality control requirements. **Figure S3.** Differentially expressed acetylated proteins were annotated based on (A) the protein domain database and (B) the KEGG pathway database.

## Data Availability

All data generated and analyzed during this study are included in this published article.

## Abbreviations

GISTs: Gastrointestinal stromal tumors
PTM: Post-translational modification
GO: Gene Ontology
KEGG: Kyoto Encyclopedia of Genes and Genomes
MS: Mass spectrometry
MH/L: Moderate and high risk/low risk
