# Supplementary material for: Quantitative proteomic analysis of aberrant expressed lysine acetylation in gastrointestinal stromal tumors
Source: Clin Proteomics. 2021 May 22;18:16. doi: 10.1186/s12014-021-09322-0 (PMC8141230; doi:10.1186/s12014-021-09322-0)
Supplement: Supplementary file 1 — Additional file 1: Figure S1. Western blotting with pan anti-succinyllysine, anti-crotonyllysine, anti-2-hydroxyisobutyryllysine, and anti-malonyllysine antibodies in 9 GIST tissue samples. Figure S2. Quality control validation of MS data. The distribution of peptide lengths identified by mass spectrometry met quality control requirements. Figure S3. Differentially expressed acetylated proteins were annotated based on (A) the protein domain database and (B) the KEGG pathway database. [file 12014_2021_9322_MOESM1_ESM.docx]

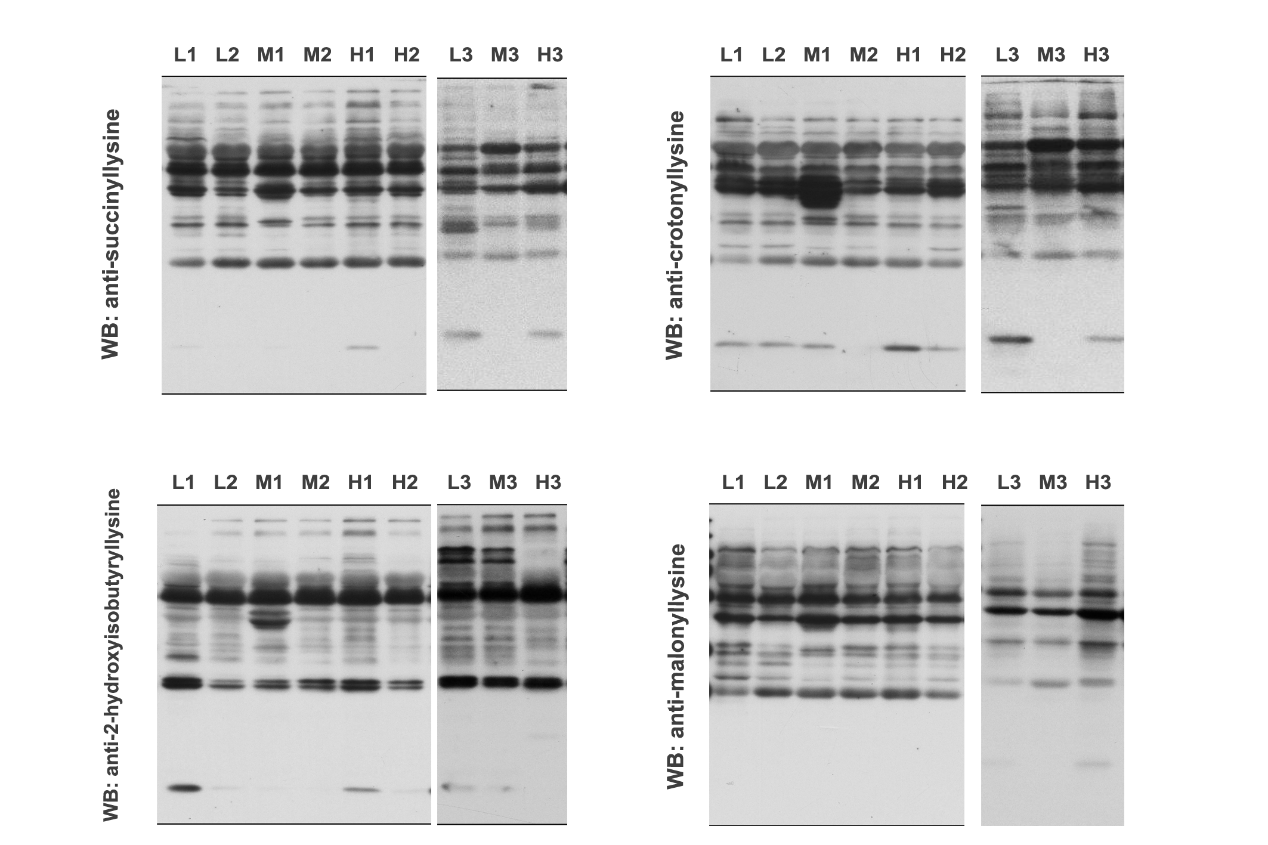


Figure S1. Western blotting with pan anti-succinyllysine, anti-crotonyllysine, anti-2-hydroxyisobutyryllysine, and anti-malonyllysine antibodies in 9 GIST tissue samples.


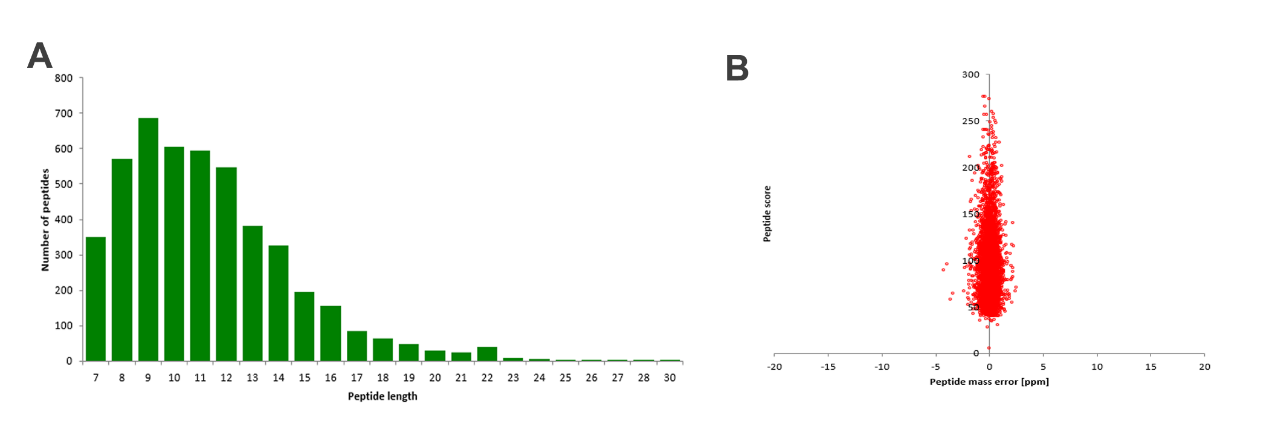
Figure S2. Quality control validation of MS data. The distribution of peptide lengths identified by mass spectrometry met quality control requirements.


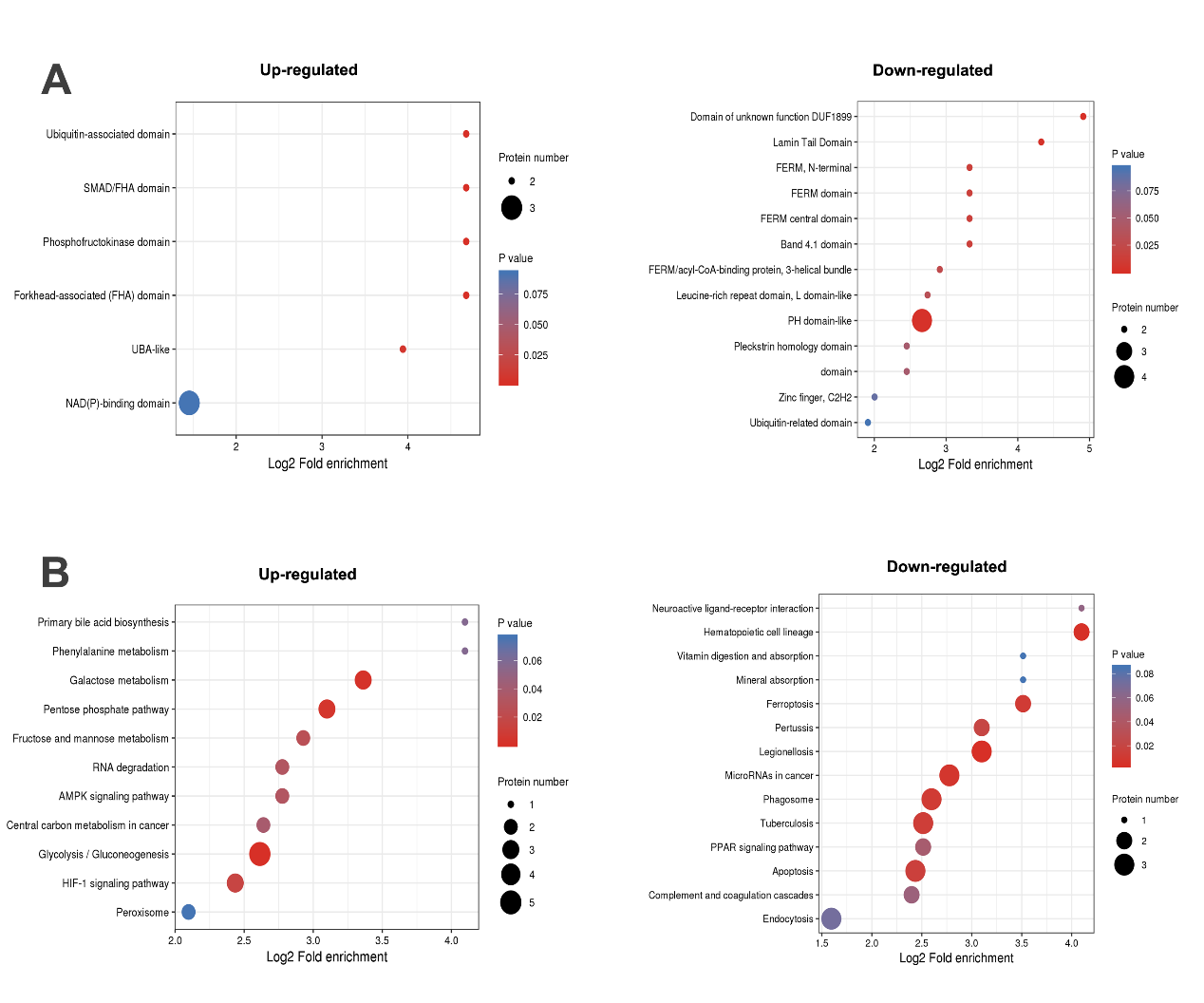


Figure S3. Differentially expressed acetylated proteins were annotated based on (A) the protein domain database and (B) the KEGG pathway database.
